# Do COVID-19 Worries, Resilience and Emotional Distress  Influence Life Satisfaction? Outcomes in Adolescents in Ecuador during the Pandemic: SEM vs. QCA

**DOI:** 10.3390/children9030439

**Published:** 2022-03-21

**Authors:** Juan Sebastián Herrera, Laura Lacomba-Trejo, Selene Valero-Moreno, Inmaculada Montoya-Castilla, Marián Pérez-Marín

**Affiliations:** 1 Faculty of Psychology, Universidad del Azuay, Av. 24 de Mayo 7-77, Cuenca 010204, Ecuador; sherrera@uazuay.edu.ec; 2 Department Personality, Assessment and Psychological Treatments, Faculty of Psychology, Universitat de Valencia, Blasco Ibáñez, 21, 46010 Valencia, Spain; laura.lacomba@uv.es (L.L.-T.); inmaculada.montoya@uv.es (I.M.-C.); 3 Department of Developmental and Educational Pscyhology, Faculty of Psychology, Universitat de Valencia, Blasco Ibáñez, 21, 46010 Valencia, Spain; selene.valero@uv.es

**Keywords:** COVID-19, adolescence, life satisfaction, resilience, psychopathology, worries about COVID-19

## Abstract

COVID-19 and the measures adopted have been a problem for society at all levels. The aim of the study was to analyze the main predictors of life satisfaction among adolescents in Ecuador during the COVID-19 pandemic. Participants were 902 adolescents from Ecuador aged between 12 and 18 years (M = 15.30; SD = 1.28). Variables such as life satisfaction, resilience, emotional symptomatology, and worries about COVID-19 were assessed. Two statistical methodologies were compared (structural equation models (SEM) and qualitative comparative analysis (QCA)) to analyze the possible influence of worries about COVID-19, resilience and emotional symptomatology towards life satisfaction. The results indicated that in both models, worries about COVID-19 were negatively related to life satisfaction. However, having a greater worry, specifically for physical health issues, was associated with better life satisfaction. SEM models indicate that depression is negatively related to life satisfaction. In QCA models, high levels of life satisfaction are explained by low levels of anxiety and depression. Thus, resilience seems to play a mediating role in life satisfaction, although this is only true for the depression variable. It is necessary to detect signs of risk in this population and strengthen resilience in them as elements that can facilitate their adequate coping with their adverse situation.

## 1. Introduction

In March 2020, the World Health Organization declared COVID-19 to be a pandemic as the number of infected and dead people worldwide increased exponentially. Due to the lack of knowledge about a cure for COVID-19, governments decreed several restrictive isolation measures [1]. Specifically in Ecuador, the state of emergency was announced on 17 March, and total confinement was declared until 13 September. As a result, schools of all educational levels were forced to close, leaving a large number of students without access to education and others having to adapt to new teaching methods [2]. 

Confinement has been a significant problem at the emotional level throughout society, increasing the presence of stress, anxiety, and depressive symptoms [3,4]. Uncertainty and quarantine have affected people’s mental health. The pandemic has prompted the use of our personal and emotional resources to try to control it. It has been experienced as a highly stressful situation due to the fear of contagion or the of contagion or of the death of oneself or a family member, loss of resources of resources, lack of supplies, change in routines, and worries and uncertainty about the future [5,6,7].

Current research reflects the emotional impact the pandemic has had and continues to have on the general population [8,9,10,11]. The emotional symptomatology most studied in the different studies carried out worldwide are anxiety, depression, and stress. The pandemic has led to an increase in anxiety, depression, and stress symptoms [8,9,10,11]. However, it has been observed that the child and adolescent population, mainly adolescents, have been the most emotionally affected group by social restriction measures [12,13], given the lower amount of personal and emotional resources to manage stressful situations [14]. Together with the general worries of the population, such as fear of contagion or death of oneself or a family member, loss of resources, shortages, habit changes, and worries and uncertainty about the future [2,5,6,7], in the case of adolescents, we must add the loss of the regular opportunity to socialize freely with their peers, an essential aspect at this point in the life cycle [13].

Generally, studies that have addressed the impact of COVID-19 on adolescents have focused on the negative impact of COVID-19, but few studies have focused on its impact from the point of view of well-being. This study approaches the measurement of well-being from different perspectives, such as hedonic and eudaimonic. The former includes the investigation of cognitive dimensions such as life satisfaction, the main variable of our study. The second includes aspects of personal growth such as resilience, also addressed in this study. Studies show that, despite existing difficulties, most people adjust emotionally in the face of adversity [15]. Therefore, it is expected that the child and adolescent population will also adapt psychologically to the pandemic [16,17]. The literature has pointed out human resilience in the face of adversity [15]. Resilience is the ability to remain emotionally stable, despite exposure to a severe stressor [18]. Thus, resilient individuals show less COVID-19-related worries, less psychopathology, and higher life satisfaction [3,15,19,20]. In this sense, it is considered that life satisfaction may be associated with better physical and emotional health outcomes [5,20], being a central element of adjustment to the COVID-19 pandemic.

To our knowledge, there are no studies based on predicting life satisfaction in the face of the COVID-19 pandemic, let alone conducted in adolescents in Ecuador. Therefore, our study aims to understand the predictors of life satisfaction among adolescents in Ecuador during confinement. The results of two statistical methodologies (structural equation models (SEM) versus models based on comparative qualitative analysis (QCA)) have been compared to analyze the possible influence of worries about COVID-19, resilience, stress, anxiety, and depression. Our hypotheses are the following: (H1) lower presence of worries about COVID-19 will be associated with higher life satisfaction; (H2) higher stress levels, anxiety, and depression will be associated with lower life satisfaction; and (H3) resilience will exert a mediating role between worries about COVID-19 and emotional symptomatology (anxiety and stress) over life satisfaction.

## 2. Materials and Methods

### 2.1. Participants 

The study included 1355 adolescents from Ecuador, of whom 902 finally participated in this research. They were between 12 and 18 years old (*M* = 15.30; *SD* = 1.28). The percentage of girls surveyed was 79.9%, while for boys, it was 19.80%, and genderqueer 0.2%. The selection criteria were (1) age between 12 and 18 years, (2) having lived during the pandemic in Ecuador, and (3) having scored less than 25% on the Oviedo Infrequency Scale (INF-OV); [21]. 

### 2.2. Measures

- Sociodemographic variables were taken through an ad hoc questionnaire.
- Resilience: The Connor–Davidson Resilience Scale (CD-RISC) [22] was used to assess resilience or the ability to cope with adversity. In the present study, we used the reduced 10-item version [23] adapted to Spanish [24]. The scale is answered from 0 to 4 (from least to most agree). Previous research shows adequate internal consistency, temporal consistency, and validity [3,23]. In our sample, internal consistency was adequate (α = 0.87).
- Life satisfaction was assessed using the Satisfaction with Life Scale (SWLS) [25] in its version adapted to Spanish [26]. This instrument comprises five items that are answered from 1 to 7, with higher values indicating greater satisfaction with life or subjective well-being. The scale has adequate internal and temporal consistency [27]. In the study sample, the SWLS showed good internal consistency (α = 0.87).
- Stress, anxiety, and depressive symptoms were assessed using the Depression, Anxiety, and Stress Scale in its reduced version adapted to Spanish (DASS-21) [28,29]. This instrument has 211 items, which results in 3 subscales (stress, anxiety, and depressive symptoms), and the scale is answered from 0 to 3 (It does not describe anything that happened to me or that I felt during the week to Yes, this happened to me frequently, or almost always). The scale assesses symptomatology in the last week quickly and briefly. The instrument has previously shown adequate psychometric properties [29,30,31] and adequate fit in Spanish-speaking samples [28,30,31]. The scale showed adequate internal consistency (stress α = 0.85; anxiety α=.83; depression α = 0.89).
- Worries about COVID-19 and its consequences were assessed using the Scale of Worries about COVID-19 and its repercussions (W-COV) (Mónaco et al., in review). The W-COV scale comprises 16 items that give rise to 3 sub-scales: health worries, economic worries, and psychosocial worries. The items are answered from 1 (Rarely) to 5 (Very frequently). In our study, the 3 factors showed acceptable reliability indices: health worries (α = 0.71), economic worries (α = 0.81), and psychosocial worries (α = 0.77).
- The infrequency of responses was assessed using the Oviedo Infrequency Scale (INF-OV) [21]. The INF-OV consists of 12 items which are answered from 1 to 5 (from “Strongly disagree” to “Strongly agree”). INF-OV assesses random, pseudorandom, or dishonest responses. Four of the scale items were selected, and participants who scored more than 25% were eliminated from the study.

### 2.3. Procedure

The assessment was conducted through the Universitat de València’s survey platform, Limesurvey, in the months of May to December 2020, after the confinement of Ecuador but during the COVID-19 pandemic. The survey was disseminated by direct contact with educational institutions in Ecuador through collaborators of the research team belonging to the University of Azuay (Cuenca, Ecuador). The questionnaire response platform includes a consent form for anonymous participation in the study. Informed consent was obtained from the parents or legal guardians of the participants, and all parties were informed of the anonymity and confidentiality of the use of their data, confirming that their participation in the study was voluntary. This study followed the guidelines of the ethical code of the Declaration of Helsinki [32] and was approved by the ethics committee of the Universitat de València (Ref. nº:1595575567385) 

### 2.4. Statistical Analysis

We performed descriptive statistics and calculated calibration values of the fsQCA were calculated. This was conducted through the program SPSS (Statistical Package for the Social Sciences, version 26, ©IBM). We then performed structural equation modelling (SEM) and fuzzy-set qualitative comparative analysis (fsQCA). Regarding SEM, we applied the estimation provided by the robust maximum likelihood (ML) estimation method in every case, which is indicated to correct for the possible absence of multivariate normality. We confirmed that the model was adequate using the Chi-square significance test and its robust correction provided by Satorra–Bentler (S-B χ²) [33,34]. EQS (Structural Equation Modeling Software, version 6.3, Bentler, 1985–2016, Multivariate Software was used for the SEM models.

To carry out the fuzzy-set qualitative comparative analysis, we transform the raw data into fuzzy-set responses. To do this, we first removed missing data and then calculated all constructs (variables) by multiplying their item scores [35,36,37]. After the above, we recalibrate the values with more than two values by considering: (0) when an observation is totally outside the set (low agreement); (0.5) when the value is neither inside nor outside the set (intermediate level of agreement); and (1) when the observation is totally inside the set (high level of agreement). When we have continuous variables or psychological factors, it is generally suggested that the three thresholds are the 10th, 50th, and 90th percentiles [38]. The fsQCA 2.5 software by Claude & Christopher (2014) recalibrated the values of resilience, life satisfaction, stress, anxiety and depressive symptoms, and concern [38]. FsQCA software (fuzzy-set qualitative comparative analysis, version 2.5, © Raging and David, 1999–2008, [39]) was used to perform fsQCA.

## 3. Results

### 3.1. Descriptive Statistics of SWLS, DASS, CD-RISC, and Worries about COVID-19

Table 1 shows the main descriptions of the study variables. Regarding the protective variables, the results indicate moderate scores in resilience capacity and moderate-high scores in life satisfaction. On the other hand, regarding the risk variables, low-moderate scores are found for depression, anxiety, and stress, being higher in the latter, and medium-high scores are found for the concern scales, with very similar scores for health, economic, and psychosocial worries.

### 3.2. Structural Equation Model (SEM)

**Figure 1:**
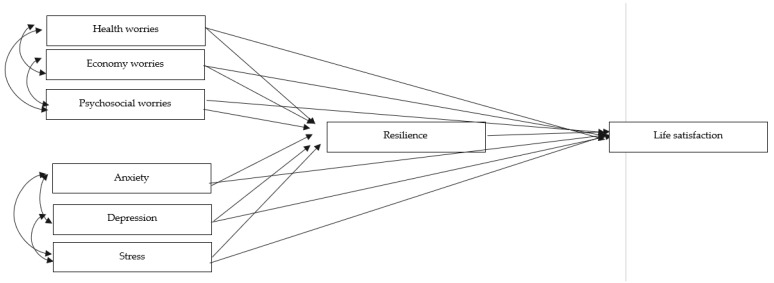
Theoretical model of causal relationships between dimensions of worries, dimensions of DASS, and resilience on life satisfaction.

**Figure 2:**
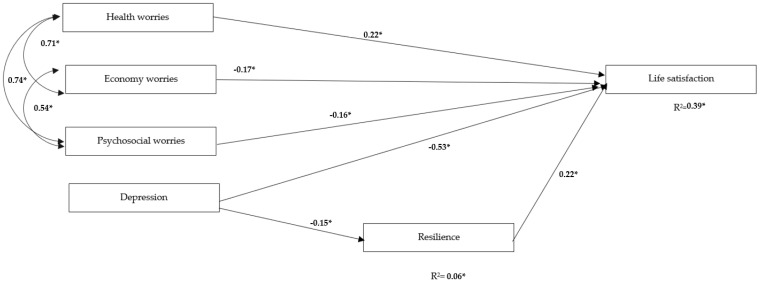
Final model of causal relationships between dimensions of worries, dimensions of DASS, and resilience on life satisfaction. * Statistically significant relationship. * = *p* ≤ 0.05; χ^2^ = 2377.43, *df* = 620, *p* ≤ 0.001; S-Bχ² = 2133.49, *df* = 620, *p* ≤ 0.001; S-Bχ²/*df* = 3,44; RMSEA = 0.05 (IC = 0.050–0.054); SRMR = 0.08; CFI = 0.87; IFI = 0.88. Note. An SEM was performed, and the items were introduced to form the scales, but due to space constraints, they are not shown in the figure.

First, the theoretical prediction model was tested (Figure 1). Figure 2 shows the final relationship-based figure. Overall, we obtained a good fit of the causal relationship model: χ^2^ = 2377.43, *df* = 620, *p* ≤ 0.001; S-Bχ² = 2133.49, *df* = 620, *p* ≤ 0.001; S-Bχ²/*df* = 3.44; RMSEA = 0.05 (IC = 0.050–0.054); SRMR = 0.08; CFI = 0.87; IFI = 0.88. Although the goodness-of-fit indices showed adequate fit, the CFI (0.87) and IFI (0.87) were below the 0.90 threshold. This may be due to the fact that the model comprises a large number of variables and indicators, especially because it is a correctly specified model [40] (note that our model comprised 620 *df*—i.e., a large number of indicators and latent variables. These authors suggested that models involving low CFI and IFI values give no real cause for concern as the RMSEA presents an appropriate adjustment. For this reason, we analyzed the goodness of fit of our SEM model by relying on the χ^2^/*df*, RMSEA, and SRMR (that indicated an appropriate adjustment of the tested model). Figure 2 shows the standardized coefficients of each relationship that have proven to be statistically significant predictors of life satisfaction. The model explained 39% (R^2^ = 0.39) of the variance, and it was found that the factor of resilience showed a statistically significant relationship in the positive (β = 0.22) and health worries (β = 0.22) and in a negative sense, economic worries (β = −0.16), psychosocial worries (β = −0.16), and depression (β = −0.53). Depression (β = −0.15) also showed a statistically significant negative relationship with resilience, explaining a 2% (R^2^ = 0.02).

The potential mediational effect of resilience on the relationship between depression and life satisfaction (Figure 2) was tested using SEM. Standardized parameter estimates are presented in Figure 2. To test the indirect effect of depression through resilience, we used an EQS function that implements Sobel’s (Sobel, 1987) test of the significance of indirect effects. The indirect effects of depression on life satisfaction (parameter estimate = 0.167; standard error = 0.023; Sobel test = 3.63) was significant at *p* < 0.05, meaning that high levels of resilience acted as a buffer against the negative impact of depression on life satisfaction.

### 3.3. Fuzzy-Set Qualitative Comparative Analysis (fsQCA)

We present the descriptive and calibration values (Table 2). 

### 3.4. Analysis of Necessity

There is no necessary condition to explain high or low levels of life satisfaction (all consistency values were below 0.90) [41] (Table 3).

### 3.5. Analysis of Necessity Sufficiency

With reference to the sufficiency analyses, we obtained the combinations of conditions that generated high and low levels of life satisfaction (Table 4). For high levels of life satisfaction, resilience was the only variable present. The frequency cut-off in the truth table was set to 1, and the consistency cut-offs were set to 0.87 based on the premise that in fsQCA, a model is informative when the consistency is around or above 0.74 [42].

High levels of life satisfaction were explained by seven combinations of causal conditions, accounting for 34% of cases (Overall Consistency = 0.79; Overall Coverage = 0.34). Low levels of life satisfaction were explained by nine combinations of causal conditions, which accounted for 44% of the cases (Overall Consistency = 0.90; Overall Coverage = 0.44) (Table 4).

Regarding high levels of life satisfaction, the most relevant pathways were: the combination of high resilience, low depression and anxiety, and low psychosocial and economic worries with high health worries (Raw coverage = 0.21; Consistency = 0.87); the combination of high resilience, low depression, and low health worries with high psychosocial and economic worries (Raw coverage = 0.18; Consistency = 0.85); and, finally, the interaction between high resilience and stress with low depression (Raw coverage = 0.18; Consistency = 0.82). These pathways explain 21%, 18%, and 18% of cases with high life satisfaction, respectively.

Regarding the prediction of low levels of life satisfaction, nine pathways were observed that explained 44% of the cases with low levels of life satisfaction (Overall consistency = 0.90; Overall coverage = 0.44). The most relevant pathways were: the interaction between low resilience and high levels of anxiety, depression, stress, and psychosocial worries (Raw coverage = 0.29; Consistency = 0.91). The second pathway was similar to the previous one (low resilience, high levels of depression, stress, and psychosocial worries) (Raw coverage = 0.28; Consistency = 0.92). The last combination was the interaction of low levels of resilience, high levels of anxiety, depression, and health worries (Raw coverage = 0.26; Consistency = 0.91). These pathways explain 29%, 28%, and 26% of cases with low levels of life satisfaction, respectively.

## 4. Discussion

Confinement has been a significant problem at an emotional level throughout society [3], increasing the presence of stress, anxiety, and depressive symptoms [4], especially for the adolescent population due to the large number of restrictions they have had to suffer in their social interactions (with the social support of peers being a substantial element in the proper development at this stage of the evolutionary cycle) [2,3].The study aims to understand the predictors of life satisfaction among adolescents in Ecuador during confinement. For this, the results of two statistical methodologies (structural equation models (SEM) versus models based on comparative qualitative analysis (QCA)) were compared to analyze the possible influence of worries about COVID-19, resilience, stress, anxiety, and depression on life satisfaction in adolescents.

Regarding (H1), the lower presence of worries about COVID-19 will be associated with higher life satisfaction, the results indicate that in both the SEM and QCA models, COVID-19 worries were negatively related to life satisfaction, as indicated by previous studies [3]. However, in our results, having a more significant concern specifically for physical health issues is shown to be associated with better life satisfaction, contrary to what was expected based on studies such as authors indicated [6] One possible explanation for this finding may be that the current pandemic situation has increased the fixation and prioritization of one’s health [44,45,46] in these times of great chaos, uncertainty, and lack of individual freedom in many of the decision-making processes of essential aspects of one’s own life (often managed by governments and global health organizations). Therefore, showing concern for one’s physical health could contribute to an increased sense of individual control over their physical health, reducing the negative impact on the well-being of the adolescent and increasing his or her life satisfaction.

On the other hand, H2 proposed that stress, anxiety, and depression would be associated with lower life satisfaction. Our data suggest that this would be the case. Moreover, the results found through the SEM models indicate that depression is negatively related to life satisfaction. At the same time, in the QCA models, it is found that high levels of life satisfaction are explained, in particular, by low levels of anxiety and depression. Therefore, as indicated by previous studies [12,19], the level of the emotional impact of COVID-19 on adolescents seems to significantly influence their ability to feel satisfied with their own life.

Lastly, H3 was approached, analyzing the mediating role of resilience on adolescent life satisfaction. The results indicate that resilience seems to play a mediating role in life satisfaction, although this only occurs significantly in the presence of the depression variable. Thus, resilience would buffer the effects of depression on life satisfaction. At the same time, the QCA models, in all the combinations that predict both high and low levels of life satisfaction, indicated that resilience is the variable that appears the most in all the significant predictions found. Therefore, our data show that resilience is a fundamental variable in the adjustment of adolescents to COVID-19, as indicated by previous studies [5,8] reducing the level of psychopathology and worries about COVID-19. 

Among the main contributions of this research would be the lack of studies focused on the subject of our work, namely, research conducted in adolescents in Latin American countries and focused on predicting positive aspects such as life satisfaction in the COVID-19 context, since most of the research on COVID-19 is based on assessing the presence of psychopathology [47]. 

On the other hand, another significant contribution is the comparison of the same results with different methodologies such as SEM and QCA models. This has made it possible to observe how variables such as psychopathology combined with resilience and worries about COVID-19 help to explain adolescent satisfaction. While the study is novel, it is not without limitations, and one of the main limitations lies in the fact that our data are cross-sectional. It would be advisable to analyze and compare adolescent adjustment to the pandemic at different points in time. However, due to the changing times we are facing, longitudinal studies may present additional difficulties in methodological rigor, since comparing different periods at the present time may involve including multiple confounding variables in their formulation and analysis. 

In our study, we had a large sample of participants; nevertheless, the results should be approached with caution in their generalization since they pertain to a single country which is Ecuador. Future research would be interesting to compare adjustment to the pandemic with other Latin American countries such as Chile, Mexico, or Colombia to analyze possible differences in psychological adjustment. In turn, these cross-cultural studies should be carried out considering the existing general cultural differences and the specific differences concerning the COVID-19 pandemic. 

Finally, the use of self-reports, despite being one of the most widely used measures in the field of psychology can lead to social desirability bias. Consequently, it would be advisable to compare these measures with those of other informants, such as family or teachers, to contrast the adolescents’ results in future studies.

## 5. Conclusions

Adolescents are a particularly vulnerable group in this situation of restriction brought by the COVID-19 pandemic, given the significant limitations they have suffered regarding their freedom of interaction and expression with their social support networks. It is necessary to detect signs of risk in this population and strengthen resilience in them as elements that can facilitate their adequate coping with the adverse situation they are experiencing, buffering its negative effects and facilitating an adequate emotional adjustment of the adolescent. Our research provides a new perspective on the emotional impact of COVID-19 in adolescents in Ecuador. The results of this study may help to understand the factors that affect the life satisfaction during the development of pandemic situation. These results will make it possible to detect protective and risk conditions for life satisfaction and to propose intervention programs that will have a positive impact on its effectiveness in improving the well-being of adolescents.

## Figures and Tables

**Table 1:** Descriptive statistics of SWLS, DASS, CD-RISC, and worries.

	CD-RISC	SWLS	DASS-21			Worries		
	Resilience	Life Satisfaction	Depression	Anxiety	Stress	Health Worries	Economy Worries	Psychosocial Worries
*M*	21.55	23.16	15.31	12.45	16.57	3.10	3.02	3.16
*SD*	8.31	6.97	11.85	19.25	10.54	0.88	0.97	0.93
*Min*	0	5	0	0	0	1	1	1
*Max*	40	35	42	42	42	5	5	5

**Table 2:** Main descriptions and calibration values.

	CD-RISC	SWLS	DASS-21			Worries		
	Resilience	Life Satisfaction	Depression	Anxiety	Stress	Health Worries	Economy Worries	Psychosocial Worries
*M*	515,080.27	3762.71	1267.04	581.42	1019.55	449.75	478.93	2064.19
*SD*	1,401,674.26	4390.41	3115.25	1918.52	2380.56	625.59	708.28	3348.85
*Min*	1	1	1	1	1	1	1	1
*Max*	9,765,625	16,807	16,384	16,384	16,384	3125	3125	15,625
Calibration values								
P10	246	80	2	1	3	12	8	18
P50	58,684	1920	54	24	96	192	144	576
P90	1,350,000	9604	4096	1296	3072	1235	1500	6400

Note: *M*: mean; *SD*: standard deviation; min: minimum; max: maximum; P10 = 10th percentile; P50 = 50th percentile; P90 = 90th percentile.

**Table 3:** Necessity analysis for life satisfaction.

	High Life Satisfaction		Low Life Satisfaction	
	Cons	Cov	Cons	Cov
High levels of health worries	0.54	0.56	0.56	0.66
Low levels of health worries	0.67	0.57	0.63	0.62
High levels of economy worries	0.51	0.54	0.56	0.68
Low levels of economy worries	0.70	0.58	0.63	0.59
High levels of psychosocial worries	0.48	0.52	0.59	0.72
Low levels of psychosocial worries	0.74	0.61	0.60	0.57
High levels of anxiety	0.30	0.46	0.45	0.78
Low levels of anxiety	0.86	0.66	0.69	0.53
High levels of depression	0.36	0.43	0.62	0.83
Low levels of depression	0.86	0.66	0.57	0.51
High levels of stress	0.287	0.49	0.40	0.81
Low levels of stress	0.89	0.56	0.75	0.54
High resilience	0.62	0.69	0.45	0.58
Low resilience	0.62	0.50	0.76	0.69

Note. Cons: consistency; Cov: coverage; Condition needed: consistency ≥ 0.90.

**Table 4:** Summary of the main sufficient conditions for the intermediate solution of life satisfaction.

Frequency Cut-Off: 1	High Life Satisfaction			Low Life Satisfaction		
	Consistency Cut-Off: 0.87			Consistency Cut-Off: 0.93		
	1	2	3	1	2	3
Health worries	**●**	**○**				**●**
Economy worries	**○**	**●**				
Psychosocial worries	**○**	**●**		**●**	**●**	
Anxiety	**○**			**●**		**●**
Depression	**○**	**○**	**○**	**●**	**●**	**●**
Stress			**●**		**●**	
Resilience	**●**	**●**	**●**	**○**	**○**	**○**
Raw coverage	0.21	0.18	0.18	0.29	0.28	0.26
Unique coverage	0.0	0.02	0.01	0.01	0.02	0.01
Consistency	0.87	0.85	0.82	0.91	0.92	0.91
Overall solution consistency			0.79			0.90
Overall solution coverage			0.34			0.44

● = presence of condition. ○ = absence of condition. Expected vector for perceived high life satisfaction: 0.0.0.0.0.0.1. (0: absent; 1: present). Expected vector for low life satisfaction: 1.1.1.1.1.1.0. using the format of [43].

## Data Availability

The datasets generated during and/or analyzed during the current study are available from the corresponding author on reasonable request.

## References

[1] European Centre for Disease Prevention and Control (2020). COVID-19. https://qap.ecdc.europa.eu/public/extensions/COVID-19/COVID-19.html.

[2] UNESCO Institute for Statistics (2020). Children Out of School, Primary. Data. https://data.worldbank.org/indicator/SE.PRM.UNER.

[3] Valero-Moreno S., Lacomba-Trejo L., Tamarit A., Pérez-Marín M., Montoya-Castilla I. (2021). Psycho-emotional adjustment in parents of adolescents: A cross-sectional and longitudinal analysis of the impact of the COVID pandemic. J. Pediatr. Nurs..

[4] Wang Y., Jing X., Han W., Jing Y., Xu L. (2020). Positive and negative affect of university and college students during COVID-19 outbreak: A network-based survey. Int. J. Public Health.

[5] Satici B., Gocet-Tekin E., Deniz M.E., Satici S.A. (2020). Adaptation of the Fear of COVID-19 Scale: Its Association with Psychological Distress and Life Satisfaction in Turkey. Int. J. Ment. Health Addict..

[6] Ahorsu D.K., Lin C.Y., Imani V., Saffari M., Griffiths M.D., Pakpour A.H. (2020). The Fear of COVID-19 Scale: Development and Initial Validation. Int. J. Ment. Health Addict..

[7] Ruiz A.L., Arcaño K.D., Pérez D.Z. (2020). La psicología como ciencia en el afrontamiento a la COVID-19: Apuntes generales. La Acad Cienc. Cuba. Int..

[8] Kämpfen F., Kohler I.V., Ciancio A., De Bruin W.B., Maurer J., Kohler H.P. (2020). Predictors of mental health during the Covid-19 pandemic in the US: Role of economic concerns, health worries and social distancing. PLoS ONE.

[9] Salari N., Hosseinian-Far A., Jalali R., Vaisi-Raygani A., Rasoulpoor S., Mohammadi M., Rasoulpoor S., Khaledi-Paveh B. (2020). Prevalence of stress, anxiety, depression among the general population during the COVID-19 pandemic: A systematic review and meta-analysis. Glob. Health.

[10] Bendau A., Petzold M.B., Pyrkosch L., Maricic L.M., Betzler F., Rogoll J., Große J., Ströhle A., Plag J. (2021). Associations between COVID-19 related media consumption and symptoms of anxiety, depression and COVID-19 related fear in the general population in Germany. Eur. Arch. Psychiatry Clin. Neurosci..

[11] Shevlin M., McBride O., Murphy J., Miller J.G., Hartman T.K., Levita L., Mason L., Martinez A.P., McKay R., Stocks T.V.A. (2020). Anxiety, depression, traumatic stress and COVID-19-related anxiety in the UK general population during the COVID-19 pandemic. BJPsych Open.

[12] Orgilés M., Morales A., Delvecchio E., Francisco R., Pedro M., Espada J.P. (2020). Coping behaviors and psychological disturbances in youth affected by the COVID-19 health crisis. Front. Psychol..

[13] Orte C., Ballester L., Nevot L. (2020). Apoyo Familiar ante el COVID-19 en España. https://preprints.scielo.org/index.php/scielo/preprint/view/297/351.

[14] Lacomba-Trejo L., Valero-Moreno S., Postigo-Zegarra S., Pérez-Marín M., Montoya-Castilla I. (2020). Ajuste familiar durante la pandemia de la COVID-19: Un estudio de diadas. Rev. Psicol. Clínica Con Niños Y Adolesc..

[15] Chen S., Bonano G.A. (2020). Psychological adjustment during the global outbreak of COVID-19: A resilience perspective. Psychol. Trauma Theory Res. Pract. Policy.

[16] Espada J.P., Orgilés M., Piqueras J.A., Morales A. (2020). Las Buenas Prácticas en la Atención Psicológica Infanto-juvenil ante el COVID-19. Clínica Y Salud.

[17] Inchausti F., García-Poveda N., Prado-Abril J., Sánchez-Reales S. (2020). Clínica y Salud. Clínica Y Salud.

[18] Bonanno G. (2004). Loss, Trauma, and Human Resilience: Have We Underestimated the Human Capacity to Thrive After Extremely Aversive Events?. Am. Psychol..

[19] Barzilay R., Moore T.M., Greenberg D.M., DiDomenico G.E., Brown L.A., White L.K., Gur R.C., Gur R.E. (2020). Resilience, COVID-19-related stress, anxiety and depression during the pandemic in a large population enriched for healthcare providers. Transl. Psychiatry.

[20] Karataş Z., Tagay Ö. (2021). The relationships between resilience of the adults affected by the covid pandemic in turkey and COVID-19 fear, meaning in life, life satisfaction, intolerance of uncertainty and hope. Pers. Individ. Dif..

[21] Fonseca-Pedrero E., Wells C., Paino M., Lemos-Giráldez S., Villazón-García Ú., Sierra S., González M.P.G.-P., Bobes J., Muñiz J. (2010). Measurement Invariance of the Reynolds Depression Adolescent Scale across Gender and Age. Int. J. Test..

[22] Connor K.M., Davidson J.R.T. (2003). Development of a new resilience scale: The Connor-Davidson resilience scale (CD-RISC). Depress. Anxiety.

[23] Campbell-Sills L., Stein M. (2007). Psychometric analysis and refinement of the Connor–Davidson Resilience Scale (CD-RISC): Validation of a 10-item measure of resilience. J. Trauma. Stress.

[24] García-León M.-Á., González-Gomez A., Robles-Ortega H., Padilla J.L., Peralta-Ramirez I. (2018). Propiedades psicométricas de la Escala de Resiliencia de Connor y Davidson (CD-RISC) en población española. An. Psicol..

[25] Diener E. (1994). Assessing subjective well-being: Progress and opportunities. Soc. Indic. Res..

[26] Vázquez C., Duque A., Hervás G. (2013). Satisfaction with life scale in a representative sample of Spanish adults: Validation and normative data. Span. J. Psychol..

[27] Bendayan R., Blanca M.J., Fernández-Baena J.F., Escobar M., Trianes M.V. (2013). New empirical evidence on the validity of the Satisfaction with Life Scale in early adolescents. Eur. J. Psychol. Assess..

[28] Fonseca-Pedrero E., Paino M., Lemos-Giráldez S., Muñiz J. (2010). Propiedades psicométricas de la escala de depresión, ansiedad y estrés versión 21 (DASS-21) eun universitarios españoles. Ansiedad Y Estrés.

[29] Lovibond P.F., Lovibond S.H. (1995). The structure of negative emotional states: Comparison of the Depression Anxiety Stress Scales (DASS) with the Beck Depression and Anxiety Inventories. Behav. Res. Ther..

[30] Bados A., Solanas A., Andrés R. (2005). Psycometric Properties of the Spanish Version of Depression, Anxiety and Stress Scales (DASS). Psicothema.

[31] Daza P., Novy D., Stanley M., Averill P. (2002). The Depression Anxiety Stress Scale-21: Spanish Translation and Validation with a Hispanic Sample. J. Psychopathol. Behav. Assess..

[32] World Medical Association (2013). World Medical Association Declaration of Helsinki: Ethical principles for medical research involving human subjects. J. Am. Med. Assoc..

[33] Hu L.-T., Bentler P.M. (1995). Evaluating Model Fit. Structural Equation Modeling. Concepts, Issues, and Application.

[34] Satorra A., Bentler P., Eye A.v., Clogg C.C. (1994). Corrections to test stadistics and standard errors in covariance structure analysis. Latents Variable Analysis: Applications to Developmental Research.

[35] Giménez-Espert M.D.C., Valero-Moreno S., Prado-Gascó V.J. (2019). Evaluation of Emotional Skills in Nursing Using Regression and QCA Models: A Transversal Study. Nurse Educ. Today.

[36] Navarro-Mateu D., Franco-Ochoa J., Valero-Moreno S., Prado-Gascó V. (2019). To Be or not to Be an Inclusive Teacher: Are Empathy and Social Dominance Relevant Factors to Positive Attitudes Towards Inclusive Education?. PLoS ONE.

[37] Villanueva L., Valero-Moreno S., Cuervo K., Prado-Gascó V.J. (2019). Sociodemographic variables, risk factors, and protective factors contributing to youth recidivism. Psicothema.

[38] Woodside A.G. (2013). Moving Beyond Multiple Regression Analysis to Algorithms: Calling for Adoption of a Paradigm Shift from Symmetric to Asymmetric Thinking in Data Analysis and Crafting Theory. J. Bus. Res..

[39] Claude R., Christopher R. (2014). fs/QCA Version 3.1b. [Computer Programme].

[40] Kenny D.A., McCoach D.B. (2003). Effect of the number of variables on measures of fit in structural equation modeling. Struct. Equ. Model..

[41] Ragin C.C. (2008). Redesigning Social Inquiry: Fuzzy Sets and Beyond.

[42] Eng S., Woodside A.G. (2012). Configural Analysis of the Drinking Man: Fuzzy-Set Qualitative Comparative Analyses. Addict. Behav..

[43] Fiss P.C. (2011). Building Better Causal Theories: A Fuzzy Set Approach to Typologies in Organizational Research. Acad. Manag. J..

[44] Orte M.C., Ballester L., Nevot-Caldentey L. (2020). Factores de riesgo infanto-juveniles durante el confinamiento por COVID-19: Revisión de medidas de prevención familiar en España. Rev. Lat. Comun. Soc..

[45] García-Fernández L., Romero-Ferreiro V., Padilla S., Lahera G., Rodriguez-Jimenez R. (2022). Different emotional profile of health care staff and general population during the COVID-19 outbreak. Psychol. Trauma. Theory Res. Pr. Policy.

[46] Piña-Ferrer L. (2020). COVID 19. A vision from the educational context in times of pandemic. Rev. Arbitr. Interdiscip. Cienc. Salud..

[47] Sandín B., Valiente R.M., García-Escalera J., Chorot P. (2020). Impacto psicológico de la pandemia de COVID-19: Efectos negativos y positivos en población española asociados al periodo de confinamiento nacional. Rev. Psicopatología Y Psicol. Clínica.

